# A combination of ascorbic acid and α-tocopherol to test the effectiveness and safety in the fragile X syndrome: study protocol for a phase II, randomized, placebo-controlled trial

**DOI:** 10.1186/1745-6215-15-345

**Published:** 2014-09-03

**Authors:** Yolanda de Diego-Otero, Rocio Calvo-Medina, Carolina Quintero-Navarro, Lourdes Sánchez-Salido, Francisco García-Guirado, Ignacio del Arco-Herrera, Isabel Fernández-Carvajal, Teresa Ferrando-Lucas, Rafaela Caballero-Andaluz, Lucia Pérez-Costillas

**Affiliations:** Unidad de Gestión Clínica de Salud Mental, Hospital Regional Universitario de Málaga, Instituto de Investigación Biomédica de Málaga (IBIMA), Hospital Civil, Pabellón 2 bajo, Plaza del Hospital Civil S/N, 29009 Málaga, Spain; Unidad de Gestión Clínica de Pediatría, Hospital Regional Universitario de Málaga, Avda Arroyo de los Angeles S/N, 29009 Málaga, Spain; Infobiotic, Calle Meridiana, 73 Blq 5-Atico B, 29018 Málaga, Spain; Unidad de Genética Molecular de la Enfermedad, Instituto de Biología y Genética Molecular (IBGM)-CSIC-Universidad de Valladolid, Calle Sanz y Fores 3, 47003 Valladolid, Spain; Servicio de Neuropediatría, Hospital Quirón, Calle Diego de Velázquez, 1, 28223 Madrid, Spain; Departamento de Psiquiatría, Facultad de Medicina, Universidad de Sevilla, Avda Sánchez Pizjuán, s/n, 41009 Sevilla, Spain; Departamento de Psiquiatría, Facultad de Medicina, Universidad de Málaga, Campus de Teatinos s/n, 29010 Málaga, Spain; Laboratorio de Investigación, Unidad de Gestión Clínica de Salud Mental, Hospital Regional Universitario de Málaga, Pabellón 6, Sótano, Hospital Civil, 29009 Málaga, Spain

**Keywords:** Antioxidants, Experimental treatment, Fragile X syndrome, Oxidative stress, Trial

## Abstract

**Background:**

Fragile X syndrome (FXS) is an inherited neurodevelopmental condition characterised by behavioural, learning disabilities, phisical and neurological symptoms. In addition, an important degree of comorbidity with autism is also present. Considered a rare disorder affecting both genders, it first becomes apparent during childhood with displays of language delay and behavioural symptoms.

Main aim: To show whether the combination of 10 mg/kg/day of ascorbic acid (vitamin C) and 10 mg/kg/day of α-tocopherol (vitamin E) reduces FXS symptoms among male patients ages 6 to 18 years compared to placebo treatment, as measured on the standardized rating scales at baseline, and after 12 and 24 weeks of treatment.

Secondary aims: To assess the safety of the treatment. To describe behavioural and cognitive changes revealed by the Developmental Behaviour Checklist Short Form (DBC-P24) and the Wechsler Intelligence Scale for Children–Revised. To describe metabolic changes revealed by blood analysis. To measure treatment impact at home and in an academic environment.

**Methods/Design:**

A phase II randomized, double-blind pilot clinical trial. Scope: male children and adolescents diagnosed with FXS, in accordance with a standardized molecular biology test, who met all the inclusion criteria and none of the exclusion criteria. Instrumentation: clinical data, blood analysis, Wechsler Intelligence Scale for Children–Revised, Conners parent and teacher rating scale scores and the DBC-P24 results will be obtained at the baseline (t0). Follow up examinations will take place at 12 weeks (t1) and 24 weeks (t2) of treatment.

**Discussion:**

A limited number of clinical trials have been carried out on children with FXS, but more are necessary as current treatment possibilities are insufficient and often provoke side effects. In the present study, we sought to overcome possible methodological problems by conducting a phase II pilot study in order to calculate the relevant statistical parameters and determine the safety of the proposed treatment. The results will provide evidence to improve hyperactivity control and reduce behavioural and learning problems using ascorbic acid (vitamin C) and α-tocopherol (vitamin E). The study protocol was approved by the Regional Government Committee for Clinical Trials in Andalusia and the Spanish agency for drugs and health products.

**Trial registration:**

ClinicalTrials.gov Identifier: NCT01329770 (29 March 2011)

## Background

Fragile X syndrome (FXS) was first described by Martin and Bell in 1943 in families with several males affected by sex-linked mental retardation [1]. It later was identified as the most common cause of inherited mental retardation [2–4]. The prevalence of FXS has been estimated at 1 in 2,500 males and 1 in 4,000 females [5, 6].

In addition to moderate to severe mental retardation, individuals with FXS exhibit macroorchidism—an elongated face, long ears, connective tissue dysplasia, hyperactivity, autistic-like and stereotypical behaviours, speech delay and increased sensory sensitivity [7, 8]. Neuropathological features of FXS are a long, thin, tortuous appearance of cortical dendritic spines, increased intracranial volume, enlarged ventricles, increased volume of selective subcortical grey-matter regions and decreased size of the posterior cerebellar vermis [9], and, in a mouse model, altered glucose metabolism [10].

The name *fragile X syndrome* came into existence upon the discovery of a fragile site in the long arm of the X chromosome detected by cytogenetic testing in a cell culture medium deprived of folic acid [11]. The fragile X mental retardation 1 (*FMR1*) gene was linked to the region (Xq27.3), and a dynamic CGG repeat expansion mutation was identified as the cause of the syndrome [12]. A full mutation with more than 200 CGG repeats causes methylation of *FMR1* and leads to transcriptional silencing of the gene [13]. It has been established that a normal range of CGG repeats varies between 6 and 55, and a CGG expansion over this range is considered abnormal. An unstable premutation allele consists of more than 55 CGG repeats, resulting in reduced levels of the fragile X mental retardation 1 protein (FMRP) encoded by *FMR1*, despite a larger quantity of its mRNA. This may be due to a compensatory mechanism derived from a translation problem in the premutated mRNA [14]. A late-onset neurodegenerative disorder that causes intention tremor, problems with coordination and balance (cerebellar ataxia) and cognitive disability has been described in males and females older than 50 to 55 years of age who are carriers of a premutation allele. This disorder is known as fragile X premutation tremor/ataxia syndrome, which is caused by an increased level of mRNA that leads to neurotoxicity in the brain [15].

The physiological effects of FMRP are not well understood, and the mechanisms that explain the pathogenesis of this syndrome remain unclear. FMRP is an mRNA binding protein and it forms complexes with additional proteins to transport target mRNA from the nucleus to the cytoplasm in microtubule-dependent movements that drive the complexes to the neurites in PC12 cells stimulated by nerve growth factor [16].

There is evidence that FXS is associated with alterations in the action of the hypothalamic-pituitary-adrenal axis [17, 18]. Recently, abnormalities in glucocorticoid secretion were shown in people with FXS and the FXS *Fmr1*-knockout mouse model [19, 20]. Also, an abnormal catecholamine content was demonstrated in this mouse model [21].

Our previous results indicate that an excess of Rac1-GTPase activation leads to nicotinamide adenine dinucleotide phosphate (NADPH) oxidase–dependent activation and high levels of free radical production in the *Fmr1-*knockout mouse brain. Elevated oxidative stress and alterations in the antioxidant system, including glutathione (GSH) decrease, are observed in the *Fmr1-*knockout brain [22]. Brain redox dysregulation alters emotion-related behaviours but leaves spatial abilities intact. Thus, a GSH deficit affects parvalbumin immunoreactive interneuron integrity and neuronal synchrony in a region- and time-specific manner, leading to behavioural phenotypes related to psychiatric disorders [23].

The central nervous system is highly sensitive to oxidative stress due to its specific anatomical and physiological characteristics. Neurons consume oxygen to produce ATP to maintain intracellular gradients of different ions (K^+^, Na^+^, Ca^2+^). Neurons are postmitotic cells that are very sensitive to oxidation. Free radicals from oxygen and nitrogen (reactive oxygen species (ROS) and reactive nitrogen species) are involved in redox regulation of several protein functions, such as glutamate transporters and neurotransmitter receptors, leading to excitotoxicity processes in the long term. This can change cellular functions and lead to long-term cell death [24].

It is well known that redox regulation is involved in many important cellular mechanisms in neurons, astrocytes and microglia, such as the activation of the mitogen-activated protein kinase (MAPK) cascade (extracellular signal-related kinase 1/2 (ERK1/2), c-Jun N-terminal kinase 1/2, p38MAPK), Ca^2+^ release and the activation of apoptotic processes [25, 26]. ROS produced by mitochondrial proteins or membrane proteins (such as NADPH oxidase activated by Rac1) have a role in physiological plasticity and may be required for normal cognitive functions [27]. An excess of ROS, however, may induce harmful changes in cellular physiology, and cells can be protected from oxidation with antioxidant processes and detoxification, such as the activation of the glutathione system. GSH plays a critical role as an antioxidant, enzyme cofactor, cysteine storage form and the major redox buffer, and it is a neuromodulator in the central nervous system. One of its most important roles is serving as a carrier/storage form for cysteine. Cysteine itself has neurotoxic effects mediated by free radical generation, which increases extracellular glutamate and triggers the overactivation of *N*-methyl-D-aspartate (NMDA) receptors. It can also serve as a neuromodulator/neurotransmitter and binds via its γ-glutamyl moiety to NMDA receptors [28]. In addition, it is thought to exert dual (agonistic and antagonistic) actions on neuronal responses mediated by NMDA receptors in the brain. GSH also serves as an endogenous nitric oxide (NO) reservoir to form *S*-nitrosoglutathione (GSNO). GSNO can release NO under certain conditions with biological effects, whereas it has a protective effect in the brain under oxidative stress conditions [29]. In addition, it is required for cell proliferation and neuronal differentiation [30].

GSH deficiency has been implicated in neurodegenerative diseases. It is a tripeptide composed of glutamate, cysteine and glycine. Cysteine is the rate-limiting substrate for the synthesis within neurons. Most neuronal cysteine uptake is mediated by sodium-dependent excitatory amino acid transporter (EAAT) systems, known as excitatory amino acid carrier 1. Previous studies have demonstrated that EAAT is regulated by redox status, leading to impaired function by glutamate accumulation in the synaptic cleft during oxidative stress [31].

Oxidative stress can activate genes that encode the enzymes of antioxidant defence or transcription factors (nuclear factor κB, activator protein 1 and nuclear factor of activated T-cells) and many other structural proteins. The increase of Ca^2+^ in neurons can activate other enzymes, including protein kinase C (PKC), phosphatase, phospholipase, neuronal nitric oxide synthase and xanthine oxidase [26, 27].

The normalization of oxidative stress can represent a new experimental target in the treatment of disorders caused by excessive production of free radicals. Oxidative stress has been found in neurological disorders, including epilepsy, Parkinson’s disease, Down syndrome, Rett syndrome, autism and Alzheimer’s disease [32]. It has been demonstrated that neuronal damage due to oxidative stress and/or hyperadrenergic states can be prevented by treatment with free radical scavengers or specific compounds that act to prevent free radical production. It has been shown that neuroprotective therapy prevents neuronal damage in neurodegenerative diseases such as Parkinson’s disease and Alzheimer’s disease [33, 34]. Nutrient deficiencies are common in attention-deficit/hyperactivity disorder (ADHD). Supplementing the diet with minerals, vitamins, essential fatty acids omega-3 and omega-6, bioflavonoids and phosphatidylserine have been demonstrated to improve ADHD symptoms [35].

Currently, the pharmacological treatment used for FXS has limited effects on the symptoms observed in patients. Stimulants of the central nervous system, such as methylphenidate, are used to treat hyperactivity, and antipsychotic drugs, such as risperidone, are used to treat aggressive behaviour. Several drugs have been used to treat anxiety, such as alprazolam and lorazepam. Patients with epilepsy have been prescribed anticonvulsive drugs. In general, a drug or drug combinations are used to treat clinical symptoms, and there are no specific drugs that prevent the appearance of the disorders [36].

High-dose vitamin E supplementation may improve insulin action and decrease plasma fasting insulin and glucose levels by decreasing cellular oxidative stress, altering membrane properties and decreasing inflammatory activity [37]. Increased vitamin E intake may enhance the endogenous cellular antioxidant defence system and reduce levels of ROS that are produced by the mitochondria. Vitamin E can also act at the cellular level independently of its antioxidant activity and may potentially contribute to improved insulin action through the inhibition of PKC [38], the decrease of intracellular levels of diacylglycerol [39] and the activation of insulin substrate protein 1 [40].

Vitamin E has also been used in children. Most clinical data are available for α-tocopherol or tocopherol esters, such as α-tocopheryl acetate. The use of vitamin E to treat diseases such as abetalipoproteinaemia [41], cystic fibrosis [42–44], β-thalassaemia, sickle cell anaemia [45], inborn errors of metabolism [46], epidermolysis bullosa [47], glucose-6-phosphate dehydrogenase deficiency [48] and focal segmental glomerulosclerosis is well documented [49]. In many studies, the rationale for dosage has not been stated and dosing regimens have not been evaluated systematically. This is demonstrated by cystic fibrosis studies in children. Doses have differed among the studies: 5.5 to 47.4 IU/kg/day, 5 to 10 mg/kg/day and 50 to 100 IU/day [50, 51].

Vitamin C has also been used intensively in sick children; most available clinical data are for ascorbic acid or antioxidant combinations. The use of vitamin C is well documented in diseases such as aphthous stomatitis [52], infant burns [53], hyperlipidaemia and arteriosclerosis [54]. An oral dosage of 2,000 mg/m^2^/day of ascorbate may modulate the generation of ROS and augment neutrophil apoptosis, which could prevent neutrophil-mediated inflammation in children. A 12-month high-dose (30 mg/kg/day) trial of oral ascorbic acid was reported to be safe and well tolerated in children ages 2 to 16 years [55]. Vitamin C was also administered, as it enhances regeneration of oxidized vitamin E. Kinetic analysis and studies of vitamin E regeneration in a protein-denaturing system revealed that ascorbate regenerates vitamin E by a nonenzymatic mechanism, whereas glutathione regenerates vitamin E enzymatically. It has been suggested that significant interaction occurs between water- and lipid-soluble molecules at the membrane–cytosol interface and that vitamin C may function *in vivo* to repair membrane-bound oxidized vitamin E [56].

## Methods/Design

We designed a clinical trial to evaluate the effects of an antioxidant combination of ascorbic acid and α-tocopherol on the clinical condition of patients with FXS. The study includes patients from age 6 years up to age 18 with diagnosed FXS. This age limit was chosen because it is within this age range that a decline in hyperactivity and behavioural symptoms may occur. The minimum duration of treatment and follow-up for these patients is to be 6 months. The symptoms most easily measured are the presence and severity of behavioural abnormalities.

Here we present a new therapeutic approach to FXS that is based on the hypothesis that an increase in free radical production and a deficit in vitamins are involved in the pathology and that this often provokes severe comorbidity. Moreover, we take into account that current treatment protocols are frequently ineffective among young children and carry a risk for important potential side effects. Thus, we propose the following goals. (1) Our main goal is to test whether the combination of 10 mg/kg/day α-tocopherol and 10 mg/kg/day ascorbic acid reduces hyperactivity and behaviour abnormalities, improving cognition among patients between 6 and 18 years of age compared to placebo treatment. (2) Secondary goals are to assess the safety of the treatment, in terms of adverse events; to describe metabolic changes resulting from the treatment, as revealed by blood test results; and to measure the impact of this treatment on the quality of family and social life.

### Design

#### Type of clinical trial

This is a phase II, double-blind, randomized clinical study. It began in December 2011 and is currently in progress.

#### Recruitment of patients

**Figure 1:**
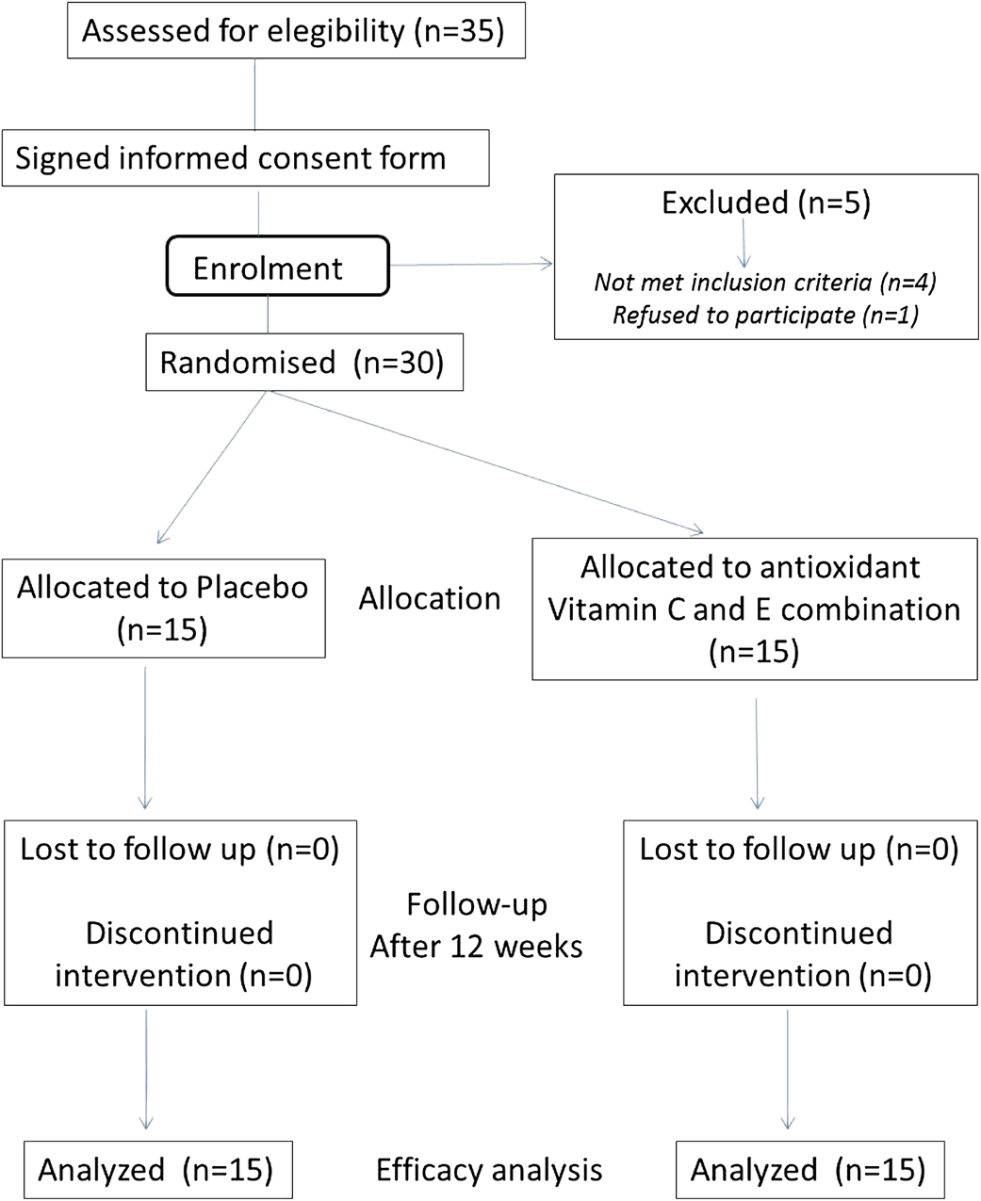
**Clinical trial flowchart.**

The patients recruited will be those diagnosed with FXS according to molecular biology test who have currently presenting symptoms. Paediatric neurologists from the Andalusian region of Spain will be informed about the clinical trial so that patients can be referred to clinics where the study will be carried out. In order to maintain double-blind conditions, the doctors responsible for patient evaluation will be derived from the pharmacy department to be allocated to one of the two study groups according to a randomization program. Informed consent will be obtained from the patients’ parents or guardians (see Figure 1).

### Study subjects

Male patients ages 6 to 18 years with a diagnosis of FXS and clinical and behavioural symptoms of the disorder will be recruited.

### Selection criteria

#### Criteria for inclusion

- Male patients ages 6 to 18 years: This is the age range during which the natural course of the condition is most exacerbated. Before the age of 6 years, hyperactivity may not yet have appeared. After age 18 years, behavioural symptoms tend to stabilize.
- Informed consent of the child’s parents or guardians and reasoned agreement with patients older than 12 years of age.
- Molecular diagnosis of FXS according to molecular biology criteria of having more than 200 CGG repeats and hypermethylation of the promoter region of the *FMR1* gene.
- Hyperactivity and behavioural symptoms of the disorder.

#### Criteria for exclusion

- Severe neurological conditions not clinically controlled.
- Unrelated neurological disorders.
- Allergy to formula components (or excipient used).

#### Randomization criteria

- Criteria set out above (age, diagnosis, consent).
- Current pharmacological treatment for behavioural symptoms.
- No contraindications due to the exclusion criteria.

Patients who fulfil these criteria will be included, randomly, in one of the two groups.

#### Randomization, blinding and assignment to treatment group

**Figure 2:**
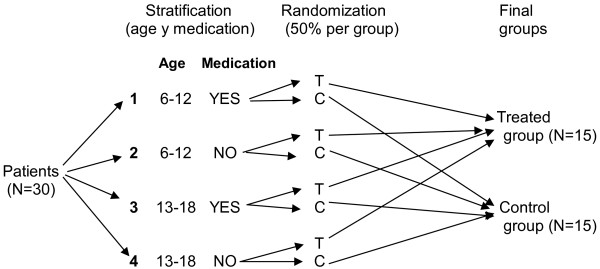
**Randomization criteria for the trial.** Randomization by blocks and stratification for confusion factors (age and concomitant medication). T: Treated group, C: Control group.

Randomization is centralized and performed immediately after the inclusion of an eligible patient. A software program will be used to ensure that allocation concealment is maintained within the pharmacy department at the Virgen de las Nieves Hospital (Granada). The randomization code will be kept concealed by the pharmacy department responsible for dispensing the corresponding medication. Randomization by blocks and stratification for confusion factors (age and concomitant medication) was performed to reduce bias (see Figure 2). Randomization to either the treatment group or the placebo group will be performed only when a patient with FXS is considered eligible to receive the medication included in this study and an informed consent form is signed by the caregiver.

#### Instrumentation

The clinical data and the results of the Wechsler Intelligence Scale for Children–Revised (WISC-R), the Conners Parent Rating Scale–Revised: Long Form (CPRS-R) [57], the Conners Teacher Rating Scale–Revised: Long Form (CTRS-R) and the Developmental Behaviour Checklist Short Form (DBC-P24) will be assessed at the baseline of the clinical condition (t0), at an intermediate time point after 12 weeks of treatment (t1) and at the end of the experimental period after 24 weeks of treatment (t2). The parents and/or guardians will be informed about the study details, and, upon receipt of their informed consent, the previously randomized medication will be provided. The medication is to be taken at the patient’s home, and a phone call follow-up is to be performed by the same neuropsychologist in charge of the evaluation at 30, 60, 120 and 150 days. Blood tests will be performed at the start (t0), intermediate time point (t1) and at the end of the experimental period (t2) for each of the 30 patients. The psychological impact of the treatment on the families concerned will be measured using the Spanish version of the Psychological General Well-Being Index–Revised (PGWBI-R).

#### Evaluations

The clinical diagnosis of FXS will be confirmed, and the Conners scale scores ascertained, so that the patient may be included in the study. Any subsequent decrease in the global score will be recorded (at t0, t1, t2). The DBC-P24, the WISC-R and metabolic changes in the blood test measurements will be recorded (at t0, t1, t2). The PGWBI-R score will be determined at t0, t1 and t2 of the study period.

#### Withdrawal of individual patients

Patients may withdraw from the study at any time for any reason and without any sanction for doing so. The researcher-collaborator, after consulting with the principal investigator and the study coordinator, may also interrupt the treatment program if the fact of continuing this treatment, in his/her opinion, is prejudicial to the patient’s welfare. If a patient withdraws or is withdrawn from the study, follow-up at day 90 should be continued whenever possible.

#### Follow-up of patients who withdraw from the study

Any patients whose treatment is withdrawn will continue to be followed until the event in question is resolved or until, in the researcher’s opinion, important changes in the patient’s health state are unlikely to occur. The follow-up reports at 15, 30, 60 and 90 days will be completed for all patients who received medication (including placebo) during this study.

#### Suspension of the study

In cases where severe adverse events related to the administration of the treatment are suspected, the study shall be interrupted and the researchers and the coordinator will decide whether to continue or otherwise. Ultimate responsibility for this decision will rest with the coordinator. The relevant clinical research ethics committees and the health-care authorities shall be informed of any decisions taken to interrupt, abandon or continue the study.

### Ethical criteria

#### Applicable regulations

The study will be carried out in accordance with the principles of the Helsinki Declaration, specifically the European Medicines Agency Committee for Proprietary Medicinal Products (EMEA/CPMP) declaration on the use of a placebo in clinical trials, and in accordance with the guideline for good clinical practice (CPMP/ICH/135/95, 17 July 1996), as well as local regulations.

#### Recruitment

The study protocol was approved by the regional clinical trial committee, the ethics committee of the University Regional Hospital (Malaga, Spain) and the Spanish Agency of Medicines and Medical Devices. Implementation of the study will start after the Spanish national health-care authorities give their formal approval. Although patients are to be informed that they are free to abandon the study at any time, we shall seek to recruit those offering the maximum probability of remaining within the study until its conclusion.

#### Informed consent for minors

After identifying candidate patients for inclusion in the clinical trial, the children will be given an oral and written explanation of the study. The parents and/or guardians will be provided with all available information and any complementary information they may require, and they will be given an information sheet so that their informed consent for the children’s participation in the trial may be obtained. Once this form has been signed, it should be given to the researcher when the child attends the clinic for the baseline evaluation (t0).

#### Liability for injury

According to the Spanish law regarding clinical trials, an insurance policy for civil liability must be subscribed to cover any injuries that may arise from the conduct of the study.

### Treatment details

#### Dosage and administration of medication

The medication used in the trial will be administered orally at the patients’ homes. The medications provided will be tocopherol acetate 10 mg/kg/day, administered in two daily doses, and ascorbic acid 10 mg/kg/day, administered in two daily doses.

#### Preparation and labelling of treatment procedures

The medications used in the trial will be prepared, labelled and stored by the pharmacy service at the Virgen de las Nieves Hospital (Granada, Spain). The active principles of the treatment group will be obtained via commercially available drugs. The placebo used will be created in the hospital’s pharmacy department and will emulate the excipients and volume of the experimental medication. Procedures for reducing the volume of medication per pack will be implemented in accordance with International Conference on Harmonisation requirements. The study coordinator will supervise all procedures applied in this respect. Double-blinding and randomization of patients in the two groups will be carried out by the pharmacy service at the Virgen de las Nieves Hospital (Granada, Spain).

#### Other medications allowed

The patients will continue taking their usual medication to control symptoms or associated comorbid pathologies. Moreover, they will continue receiving any preexisting psychological or educational therapies. In addition, they will continue taking any medication prescribed for any other concurrent illness prior to their entering the study.

### Specific methods

#### Evaluation of effectiveness

The clinical evaluation of the patients will be carried out by utilising the CPRS-R) [57] and the CTRS-R [58]. These scales have been validated for the study of hyperactivity in children. The Conners score is applied by means of a structured questionnaire with multiple informants (generally parents and teachers) who assess the child’s behaviour over a period of at least 1 month. The translation into Spanish and its adaptation to local conditions were previously validated [59].

#### Measurement instruments

The Spanish version of the CPRS-R will be used. Scoring on this scale ranges from 0 to 100 points (T-score). A T-score of 50 or more on the CPRS-R at t0 will be considered a cutoff for the inclusion of patients in the study.

The Spanish version of the CTRS-R will be used. Scoring on this scale ranges from 0 to 100 points.

The DBC-P24 will be used [60]. The Spanish version of the DBC-P24 (90) was used to identify the degree of behavioural difficulties among participants. Each behavioural description is scored with a 0, 1 or 2 rating, where 0 = ‘not true as far as you know’, 1 = ‘somewhat or sometimes true’ and 2 = ‘very true or often true’. The tally provides a total score.

Completed PGWBI-R (Spanish version) forms will be obtained from parents at the beginning and the end of the study in order to assess the psychological repercussions of the treatment on family life. This scale reflects subjective feelings and psychological well-being (or otherwise) during the previous week [61].

Oxidative stress status in blood will be assessed at t0, t1 and t2 to detect metabolic changes that may be related to the effectiveness of the treatment. Qualitative changes in the metabolism and antioxidant levels will be evaluated.

#### Safety evaluation

The following evaluations will be performed before and after the treatment:

1. Haematology: haemoglobin, haematocrit, red blood cells, white blood cells and platelets
2. Biochemistry: creatine, blood urea nitrogen, glucose, uric acid, sodium, potassium, calcium, phosphate, chloride, total protein, albumin, triglycerides, total cholesterol, high-density lipoprotein, total bilirubin, aspartate aminotransferase (serum glutamic oxaloacetic transaminase), alanine transaminase (ALT; serum glutamic pyruvic transaminase), γ-glutamyl transpeptidase, alkaline phosphatase, cAMP, glutamic acid and pyruvic acid
3. Hypothalamic-pituitary-adrenal axis (adrenaline, noradrenaline, dopamine, cortisol, adrenocorticotropic hormone)
4. Other tests
  - Hormones (luteinizing hormone, follicle-stimulating hormone, growth hormone, thyroid-stimulating hormone and thyroid hormone)
  - Neurotransmitters (serotonin, γ-aminobutyric acid (GABA))
  - cAMP
  - Vitamins (vitamins A, C and E)
  - Ceruloplasmin, albumin and transferrin
  - Minerals (selenium, zinc, copper and manganese)
  - amino acids in serum
5. Analysis of urine density, pH, protein, glucose, ketone bodies, bilirubin, blood, nitrite, urobilinogen, leukocytes, urinary sediment, sodium, chlorine and potassium

The following medical parameters will be evaluated in the enrolled patients:

1. Heart symptoms: mitral valve prolapse
2. Neurological symptoms: epilepsy
3. Motor symptoms: hypotonia, fine and gross motor delay, stereotypes and hyperextensible finger joints
4. Morphological features: macroorchidism, scoliosis, hyperpigmentation of the skin, otitis, strabismus and macrocephaly

#### Reporting of adverse events

Any adverse events reported spontaneously by the subject or observed by the researcher or the research team will be recorded on the form designed for this purpose. The researcher will classify the intensity of said adverse events in accordance with the following scale:

1. *Mild*: some discomfort, but not such as to interrupt normal daily activity
2. *Moderate*: sufficient discomfort to reduce or notably affect normal daily activity
3. *Severe*: causing incapacity to work or perform normal daily activities

The periodicity of the event shall be classified in accordance with the following scale:

1. *Single occurrence*: just one event of limited duration
2. *Intermittent*: various episodes of an event, each of limited duration
3. *Persistent, unlimited*: an event that has persisted over time and is of indefinite duration

For each adverse event, its relation to the medication taken (definitive, probable, possible, improbable, none), in the researcher’s opinion, as well as any action taken as a result, will be recorded on the data collection form. The occurrence of an adverse event that is fatal, potentially fatal or incapacitating, or that requires or prolongs hospitalization, or that provokes severe congenital anomalies will be recorded as a ‘severe’ adverse event (SAE).

All SAEs and unexpected adverse pharmacological reactions, defined as adverse events whose nature or intensity is not in accordance with any adverse event expected, will be reported by the researcher to the study coordinator by telephone, mail or fax as soon as is reasonably possible, but in any case within 24 hours of occurrence.

#### Follow-up after occurrence of an adverse event

All adverse events will be observed until their remission or stabilization. Depending on the circumstances, this observation might necessitate evaluation by referral to the patient’s general practitioner and/or specialist.

### Procedures and control

#### Selection of subjects

Patients diagnosed with FXS will be included in a preliminary ‘potential subjects’ group. Before any selection activity is undertaken, a written informed consent form, signed and dated, is to be obtained from each parent or guardian. The patient’s parents will be informed, before any action is taken, of the purposes of the study, and any doubts expressed will be answered. It must be stressed that patients have the unconditional right to withdraw from the study at any time. They will be asked to return to the health-care clinic to begin the study procedure.

#### Study periods

*Entry into the study (t0)*: The child’s parents and/or guardians agree to be included and sign the informed consent to enter into the clinical active phase of the study. The CPRS-R, CTRS-R, WSIC-R and DBC-P24 results will be assessed, taking into account that a score of at least 50 on the CPRS-R is a prerequisite for entry into the study. The baseline blood and urine analyses will be carried out for included patients. The PGWBI for the parents and guardians will be calculated.

*Intermediate period (12 weeks, t1)*: The CPRS-R, CTRS-R, WSIC-R and DBC-P24 scores will be calculated. The t1 blood and urine analyses will be carried out for included patients. The PGWBI scores for the parents and guardians will be calculated.

*End of study period (24 weeks, t2)*: The CPRS-R, CTRS-R, WSIC-R and also DBC-P24 are calculated. The T2 blood and urine analyses will be carried out for patients included in the study. The PGWBI for the parents/guardians will be calculated.

### Data analysis

#### Calculation of statistical power, establishment of sample size and safety

The sample size will be established by means of a pilot scheme based on a phase II effectiveness trial with 30 patients monitored over the course of 6 months for a level of significance of 0.05 and a statistical power of 0.8, taking the least favourable case. On the basis of the prevalence of FXS, 13 patients per group (26 in total) will be needed. This sample size will then be overdimensioned to allow for a possible dropout rate of 10%, and so the minimum sample size is to be calculated as *N* = 30 (15 patients per group).

#### General considerations

A descriptive statistical analysis will be performed using statistics of central trend and dispersion for the quantitative variables and frequency distributions for the categorical variables carried out separately for the experimental and control groups. The baseline variables will be compared using these techniques. A flow diagram will be drawn to show the sequence from the initially eligible population to those excluded from the study (refusals, dropouts, lost to follow-up and so forth) in accordance with the criteria in the CONSORT guidelines.

A comparison will be made of between-group differences in the initial and final measurements for the diverse elements of the CPRS-R, CTRS-R and DBC-P24 using unpaired nonparametric tests. For the final within-group comparison, paired nonparametric tests will be used for nonparametric repeated measures with adjustments for baseline imbalances and scores. The same analysis will be performed for the index of psychological well-being to measure the repercussions of the treatment on family life.

The principal outcome variable (POV) will be taken as the change in global score on the Conners scales, adjusted for the baseline values. A simple linear regression model will be constructed for this POV, and the level of significance will be set at *P* < 0.05. Subsequently, a multiple linear regression model will be created, including the variable ‘Experimental/Control Group’ and adjusting for any baseline variables that may be unbalanced. Statistical adjustment will be performed by means of a multivariate model including the variable group (experimental and control) and concomitant treatment (yes or no). The safety of the treatment will be described in terms of the percentage of adverse effects.

## Discussion

FXS is considered a rare neurodevelopmental disorder, although different rates of prevalence have been reported in current studies [2, 5]. The condition is seldom diagnosed in Spain due to ignorance of its existence and characteristics [5]. Until very recently, FXS was recognized as such only for the most severe cases, in which there was an important degree of functional limitation and autism very evident. Although this situation in Spain is changing, FXS is still considered an uncommon disease.

Few clinical trials have been carried out with children with FXS, because, in addition to the normal difficulties arising in this kind of study (with adults), legal considerations must be borne in mind due to the necessity to protect minors. Nevertheless, such studies are clearly needed, and several are already ongoing or finished (see Table 1).

**Table 1:** **Double-blind, randomized, placebo-controlled crossover studies of treatment of children with fragile X syndrome** ^**a**^

Clinical trial registration number	Type	Compound	Population	Target	Status	Promoter	Results (reported or pending)
NCT01894958	Phase II multicentre	NNZ-2566	Adolescent and adult males	NMDA antagonists	Recruiting	Neuren Pharmaceuticals (USA)	Pending
NCT01725152	Phase II single centre	Ganaxolone	Adolescents and children	GABA-A agonist	Recruiting	Marinus Pharmaceuticals (USA)	Pending
NCT02126995	Phase II single centre	Metadoxine (MG01CI)	Adults and adolescents	Ion pair of pyridoxine (vitamin 6)	Not yet recruiting	Alcobra Pharma (USA)	Pending
NCT01474746	Phase II single centre	Sertraline	Children	Selective serotonin reuptake inhibitors	Recruiting	University of California, Davis (USA)	Pending
NCT01911455	Phase II and III Multicentre	Acamprosate	Adolescents and children	NMDA receptor modulators	Recruiting	Children’s Hospital Medical Center (Cincinnati, OH, USA)	Pending
NCT01357239	Phase II multicentre	Mavoglurant (AFQ056)	Adolescents and adults	mGlur5 antagonist	Terminated	Novartis (Basel, Switzerland)	[62]
NCT01348087							
NCT01013480	Phase II multicentre	Arbaclofen (STX209)	Adolescents and adults	GABA-B agonist	Terminated	Seaside Therapeutics (USA)	[63]
							[64]
NCT01053156	Phase II single centre	Minocycline	Adolescents and children	Antibiotic	Completed	University of California, Davis (USA)	[65]
NCT01015430	Phase II multicentre	Basimglurant (RO4917523)	Adults	mGlur5 antagonist	Completed	Hoffmann-La Roche	Pending
NCT00895752	Phase IV single centre	Riluzole	Adults	Inhibitor of glutamate release	Completed	Indiana University (USA)	[66]
NCT01254045	Phase II single centre	Oxytocin	Adolescents and adults	Social brain neuropeptides	Completed	Stanford University (USA)	[67]
NCT01120626	Open label	Donepezil	Adolescents and children	Cholinergic drug	Completed	Stanford University (USA)	[68]

^a^GABA, γ-aminobutyric acid; GABA-B, γ-aminobutyric acid; mGluR5, metabotropic glutamate receptor 5; NMDA, *N*-methyl-D-aspartate.

FMRP is an mRNA-binding protein that is important for mRNA transport, mRNA stabilization and translation regulation of mRNA into protein at the synapse [69]. FMRP is also a factor in the regulation of brain oxidative stress, so, in the absence of FMRP, there is hyperactivation of Rac1-GTPase-dependent NADPH-oxidase signalling. These alterations lead to an excess of free radical production that, in the long term, produces oxidative stress, which is a crucial factor in the central nervous system that disrupts neuron, astrocyte and microglia communication [32]. Evidence of oxidative stress in FXS is manifested by high levels of oxidised proteins, lipid peroxidation end products, formation of carbonyls and oxidative alteration of the glutathione system in the brain of the *Fmr1*-knockout mouse model [21].

Since 1983, it has been indicated that vitamins can improve FXS patients’ cognitive status. The first vitamin used for the treatment of the FXS was folic acid. Several publications describe the efficacy and safety of treatment with folic acid in people with FXS [70–73].

Two double-blind trials have assessed the safety and efficacy of L-acetylcarnitine (LAC) in boys with FXS and an additional diagnosis of ADHD. Both of the trials were randomized, placebo-controlled studies with a parallel-group design. The first study included 20 patients and compared LAC with a dose of 100 mg/kg/day versus placebo, and the investigators found no significant difference between them based on the WISC-R or the Bender-Gestalt tests and Conners questionnaires completed by teachers. However, the results CPRS-R and CTRS-R questionnaires completed by parents and teachers at school showed a significant reduction (Hedges *g* effect size = −3.94, SE = 0.91) of hyperactive behaviour at the end of the study in the LAC-treated subjects [74]. The second study, classified by the authors as a phase II study, involved eight centres in three European countries. The investigators compared LAC at doses of 20-50 mg/kg/day versus placebo in 63 patients. A statistically significant stronger reduction of hyperactivity and improvement of social behaviour was observed in patients treated with LAC compared with the placebo group, based on the results from the Conners Global Index–Parents (CGI-P) (Hedges *g* effect size = −0.30; SE = 0.28) and the Vineland Adaptive Behaviour Scales Survey Form (Hedges *g* effect size = 0.52 and 0.65, SE = 0.29 and 0.29). They also reported no significant side effects in the LAC group [75].

In one phase II parallel, randomized, double-blind, placebo-controlled clinical trial of 4 weeks’ duration, researchers assessed the efficacy and safety of the ampakine compound CX516 versus placebo in 49 patients with FXS, 27 of whom were taking concomitant psychoactive medication. Twenty-one of the patients had an additional diagnosis of autism, and four had autism spectrum disorder. The results of that study showed no significant improvement in memory, the primary outcome measured, or in secondary measurements of language, attention/executive function, behaviour and overall functioning in CX516-treated subjects compared to placebo. There were minimal side effects, no significant changes in safety parameters and no SAEs [76].

There are also enhanced, abnormal epileptiform discharges consistent with an enhanced rate of clinical seizures in FXS patients, as well as auditory-dependent seizures in the mouse model. There are several studies regarding the use of tocopherol to control seizures in animal models and humans [77].

Excessive metabotropic glutamate receptor 5 (mGluR5) signalling has been proposed to be responsible for the psychiatric and neurological symptoms of FXS, including cognitive deficits, seizures, anxiety, perseverative movements and social deficits. It has been documented that a 50% reduction in mGluR5 expression in the *Fmr1-*knockout mouse rescued most FXS abnormalities, including altered ocular dominance plasticity, increased density of dendritic spines on cortical pyramidal neurons, increased basal protein synthesis in the hippocampus, exaggerated inhibitory avoidance extinction, audiogenic seizures and accelerated body growth. However, macroorchidism was not rescued. The results obtained with the use of mGluR5 antagonists in animal models of FXS further support the mGluR theory. 2-Methyl-6-phenylethynyl pyridine hydrochloride (MPEP) is a potent, highly selective antagonist of mGluR5 receptors. MPEP is toxic to humans, so other mGluR5 antagonists, such as fenobam and mavoglurant (AFQ056), have been studied in FXS. Fenobam was found to be safe in a single-dose trial in 12 adults with FXS. There were improvements in hyperactivity and anxiety, and 50% showed at least a 20% improvement in prepulse inhibition [78].

In a recent randomized, double-blind, two-treatment, two-period, crossover clinical trial of 30 male FXS patients ages 18 to 35 years, investigators examined whether a receptor subtype-selective inhibitor of mGluR5, AFQ056, improves the behavioural symptoms of FXS. The results indicated no significant effects of treatment on the primary outcome measure, the Aberrant Behaviour Checklist–Community Edition (ABC-C) score, at day 19 or 20 of treatment. Surprisingly, owing to an epigenetic effect, seven patients with full *FMR1* promoter methylation and no detectable *FMR1* mRNA, as measured with the ABC-C, improved significantly more after AFQ056 treatment than with placebo (*P* < 0.001). Twenty-four of thirty patients experienced adverse events, which were mostly mild to moderately severe fatigue or headache [79]. A pilot add-on trial was conducted to evaluate the safety and efficacy of lithium in humans with FXS. It was hypothesized that the absence of FMRP disrupts regulation of group 1 mGluR- and mGluR5-dependent translation in dendrites. Lithium was found to reduce mGluR-activated translation and reverse phenotypes in the *dfxr*-mutant fly and *fmr1*-knockout mouse. The results indicated that lithium is well tolerated and provides functional benefits in FXS, possibly by modifying the underlying neural defect [80].

GABA_B_ receptor agonists such as baclofen inhibit both presynaptic release of glutamate and postsynaptic transmission and/or intracellular signalling downstream from mGluR5. A double-blind, placebo-controlled crossover trial of arbaclofen has been completed in multiple centres. It included more than 150 individuals with FXS (ages 6 years and older). The preliminary safety and efficacy results are positive, with improvement in the Clinical Global Impression improvement scale in those with the most severe baseline ratings, but the trial was terminated in 2013 [63, 64]. The GABAergic system is also dysregulated in FXS, and GABA is a major inhibitory neurotransmitter receptor in the brain which is involved in anxiety, depression, epilepsy, insomnia, learning and memory. GABA-mediated inhibition is important in the prevention of seizures [81].

The results of an open-label riluzole clinical trial in FXS were published recently. Glutamatergic dysregulation was implicated in the pathophysiology of FXS. Riluzole was hypothesized to have an inhibitory effect on glutamate release, block excitotoxic effects of glutamate and potentiate postsynaptic GABA_A_ receptor function. ERK activation is known to be delayed in humans with FXS and in FXS-knockout animal models. Correction of delayed ERK activation is a potential biomarker of treatment response in FXS. The results of that 6-week open-label study of six adults with FXS show that riluzole (100 mg/day) was not associated with a significant clinical improvement, despite uniform correction of peripheral ERK activation [66].

In another study, the use of valproic acid (VPA) was investigated in an attempt to identify drugs capable of restoring the activity of the *FMR1* gene. VPA is a well-known antiepileptic drug also used as a mood stabilizer and in migraine therapy. The VPA treatment was capable of producing an adaptive behaviour improvement (defined as the performance of daily activities required for personal and social competence) due to a significant reduction in hyperactivity [82].

Another research group conducted a 4-week, randomized, double-blind, placebo-controlled, crossover study with either 3 mg/day of melatonin or placebo given to participants for 2 weeks and then alternated for another 2 weeks. The results of this study support the efficacy and tolerability of melatonin treatment for sleep problems in children with FXS [83].

Minocycline, a widely used antibiotic used to treat acne and skin infections, is another promising drug that may target FXS core symptoms. Minocycline inhibits matrix metalloproteinase 9 (MMP-9) and reduces inflammation in the central nervous system. MMP-9 is elevated in FXS. When minocycline was administered to *Fmr1-*knockout mice, their hippocampal neurons exhibited mature dendritic spines, and, behaviourally, they showed decreased anxiety and improved exploration skills. Off-label use of minocycline to treat 50 individuals with FXS resulted in two-thirds of families noticing positive language and behavioural improvements in their children while on the medication. The most common reported side effect was gastrointestinal difficulty, including loss of appetite [84]. Paribello *et al*. reported beneficial effects on the CGI and the Aberrant Behaviour Checklist in an open trial of minocycline involving 20 patients with FXS who were 13 years of age or older [85].

The first trial of acamprosate, a drug with putative mGluR5 antagonism, was reported in three adults with FXS and autism. Medical records describing open-label treatment with acamprosate in three patients with FXS and a comorbid diagnosis of autistic disorder were reviewed. In all three patients, acamprosate was associated with improved linguistic communication. Three patients received acamprosate over a mean 21.3 weeks of treatment They showed a global clinical benefit and a marked communication improvement was unexpected and has potential implications for the treatment of FXS [86].

In an open-label 12-week trial of aripiprazole in 12 persons with FXS ages 6 to 25 years (mean age, 14.3 years) who were free of concomitant psychoactive drugs, the results indicated a ≥25% improvement on the Aberrant Behaviour Checklist irritability subscale in 10 (87%) of 12 patients. Aripiprazole is generally safe and well tolerated and is associated with significant improvements in irritable behaviour [87].

To assess positive antioxidant effects versus placebo, a one-way crossover study was selected due to the impossibility of abolishing a carryover of treatment effect from the first period of treatment to the next. A ‘carryover effect’ means that the observed difference between treatments depends upon the order in which the treatments were received; hence the estimated overall treatment effect will be affected (usually underestimated, leading to a bias towards the null) [88].

Orally administered antioxidants such as tocopherol and ascorbic acid have been used as a nutritional supplement, and it they are considered safe even for children. Tocopherol is contraindicated in cases of vitamin K deficiency caused by malabsorption or anticoagulant therapy. The US Food and Drug Administration’s recommended daily dose is 10 mg/day, but the safety dose is considered to be 800 mg/day [89].

Vitamin E (α-tocopherol) is a liposoluble vitamin with a wide therapeutic margin. In clinical and pharmacological trials, it has been shown to have interesting properties, participating in oxidative deamination, transamination and decarboxylation. It also participates in the decarboxylation of glutamic acid to GABA, from levodopamine to dopamine and from 5-hydroxytrytophan to serotonin. It presents anticonvulsant properties and seems to exercise a neuroprotective and antitoxic effect. It can be administered to children and has been authorized for use to treat children with alterations in character, language and behaviour; learning difficulties; delayed learning to walk; convulsive illnesses; intoxication of the central nervous system; trembling; and Parkinson’s disease. The dosage provided may vary widely, as renal elimination ensures that its toxicity is minimal [90].

A pilot study was designed to evaluate the safety of a novel micelle formulation (CF-1) of fat-soluble nutrients and antioxidants and to determine its efficacy in improving plasma levels of these compounds and reducing inflammatory markers in induced sputum. The novel CF-1 formulation safely and effectively increased plasma levels of important fat-soluble nutrients and antioxidants. In addition, improvements in antioxidant plasma levels were associated with reductions in airway inflammation in cystic fibrosis patients [91]. The effect of α-tocopherol and ascorbic acid on ALT levels and insulin resistance has been evaluated in children with nonalcoholic fatty liver disease [92].

The follow-up period of 6 months in our present trial is based on previous trials and a minimum period of improving symptoms, such as behaviour and antioxidant status. We believe that if the patient enters the study with a Conners T-score > 50, it will be easier to identify significant differences, with the symptoms being controlled to a greater extent, and more quickly, in the experimental group than in the control group.

The reason for performing blood and urine tests in our experimental group at t0, t1 and t2 is to corroborate the general safety of the treatment and to improve antioxidant blood status, as well as to reveal any alterations occurring in these parameters as a result of the medication administered. Nevertheless, it seems reasonable to support the results of the clinical evaluation with objective data such as blood tests. It is also important to assess the repercussions on family life (which tend to be greatly impaired in severe cases) of an improvement in the control of FXS symptoms among children. The PGWBI reflects psychological well-being (or otherwise); it is based on theories of evaluation of the domestic environment and is an appropriate means of determining the distortion produced by FXS within the household. We have no doubt that a direct correlation will be found between the children’s symptoms and psychological well-being within the family home.

The combined application of these three measurement methods—namely, the objectification of FXS symptoms and incapacity, the assessment of metabolic blood and urine changes and the evaluation of stress within the family—will enable us to reach an objective judgment of the effectiveness of the treatment being tested.

## Conclusion

Treatment for FXS continues to present important shortcomings and further clinical trials are necessary in this respect, especially among children showing more severe symptoms.

## Trial status

This trial was designed in 2010. The protocol passed through multiple amendments. Final approval was obtained at the end of 2010. The expected duration of the study is 4 years. Also published at https://www.clinicaltrialsregister.eu/ctr-search/trial/2009-017837-23/ES.

## Abbreviations

ADHD: Attention-deficit/hyperactivity disorder
CONSORT: Consolidated Standards of Reporting Trials
FDA: Food and Drug Administration
GABA: γ-aminobutyric acid
NMDA: *N*-methyl-D-aspartate
PGWBI: Psychological General Well-Being Index
POV: Principal outcome variable
SAE: Severe adverse event
CPRS-R: Conners Parent Rating Scale–Revised: Long Form
CTRS-R: Conners Teacher Rating Scale–Revised
DBC-P: Developmental Behaviour Checklist for Parents
WISC-R: Wechsler Intelligence Scale for Children–Revised.
